# Quilting suture is better than conventional suture with drain in preventing seroma formation at pectoral area after mastectomy

**DOI:** 10.1186/s12893-020-00725-8

**Published:** 2020-04-06

**Authors:** Yuhui Wu, Shouman Wang, Jian Hai, Jie Mao, Xue Dong, Zhi Xiao

**Affiliations:** 1 Department of Breast Surgery, Xiangya Hospital, Central South University, 87 Xiangya Road, Changsha, Hunan P. R. China 410008; 2 Clinical Research Center For Breast Cancer Control and Prevention In Human Province, Changsha, P. R. China

**Keywords:** Quilting suture, Seroma, Mastectomy

## Abstract

**Background:**

The aim of this study was to compare quilting suture with conventional suture on the formation of seroma at pectoral area after mastectomy (ME) with sentinel lymph nodes biopsy (SLN) or axillary lymph nodes dissection (ALND) for breast cancer.

**Methods:**

Two hundred thirty-five consecutive breast cancer patients were retrospectively analyzed. The primary outcome was the incidence of Grade 2 or Grade 3 seroma at anterior pectoral area within 1 month postoperatively. We categorized seroma into early or late seroma according to the drainage removal time. Cox regression was used for analysis.

**Results:**

The incidence of Grade 2 and 3 seroma was significantly higher in the conventional suture group compared with that in the quilting suture group (19.3% vs. 9.5%, *p* = 0.032), which was attributed to the late seroma in Grade 2 and 3. Quilting suture was associated with longer time for fixing flaps compared with that of conventional suture (504.7 s vs. 109.1 s, *p* < 0.001), but with less volume of drainage. Old age, high body mass index and conventional suture were independently risk factors for Grade 2 and 3 seroma.

**Conclusions:**

Quilting suture decreased the incidence of Grade 2 and 3 seroma at pectoral area within 1 month after mastectomy, especially the late seroma in Grade 2 and 3.

## Background

Mastectomy is a standard and the most popular treatment for breast cancer in China [1]. Wound seroma in the dead space beneath skin flaps is the most common complication after mastectomy for breast cancer. The incidence varies from 15.5 to 92% reportedly, depending on various risk factors such as age, body mass index (BMI), type of surgerical procedure, drainage system and dissection instrument [2–5].

Although many patients with seroma were asymptomatic, some experienced pain, paraesthesia and even persistent aspiration of fluid for months. The traumatic aspiration might increase the incidence of surgical site infection, clinic visit, and mental stress of patients. Many kind of techniques have been applied to reduce the seroma after mastectomy, such as suction drainage, shoulder immobilization, quilting sutures, fibrin sealants and thrombin sealants [6–10]. The most commonly used method of lowering seroma formation was to place drainage system postoperatively, however there were still some controversies about the number of drainage tubes we need to use, drain location, type of drainage system, drainage removal time, and no drainage alternatives [5, 8]. Another problem is that drainage is associated with patient discomfort, increased infection and longer hospital stay. Furthermore, seroma might recurrently occur after the drainage removal.

Several studies applied quilting suture to omit the drainage after mastectomy for breast cancer. Some found a significant decrease of seroma incidence [8, 11, 12], but some didn’t [13, 14]. The quilting suture technique is to suture the skin flaps to the underlying musculature in order to reduce the dead space. This technique was applied firstly in breast reconstruction and then recently extended to mastectomy for breast cancer to reduce dead space and seroma formation [15–17]. Some studies evaluated the concomitant use of both quilting suture and drainage to reduce the incidence of seroma, while others used quilting suture only to prevent the formation of seroma in pectoral area but with drain for axilla, and some even omit all the drains after mastectomy and axillary lymph nodes dissection with skin-flap quilting [11, 12, 18, 19].

In our daily practice, we have noticed that the most common place of developing seroma postoperatively are the axilla as well as the medial and inferior border of the dead space created by the dissection at the pectoral area. In this study, we retained the drain for axilla, and compared the effect of quilting suture applied at medial and inferior border of the dead space at the pectoral area with conventional suture on seroma formation in women who underwent mastectomy with or without axillary lymph nodes dissection for breast cancer.

## Methods

### Patients

We retrospectively analyzed 235 consecutive breast cancer patients who underwent mastectomy plus SLN or ALND between March 2016 and January 2019 in the Breast Surgery Department of Xiangya Hospital, Central South University, Changsha City, R.P. China. Patients treated with bilateral breast mastectomy surgery, breast-conserving surgery, breast reconstruction surgery, skin grafting, and patients with scleroderma or systemic lupus erythematosus were excluded. All patients were local citizens older than 18 years and were first diagnosed as breast invasive carcinoma or ductal carcinoma in situ. During March 2016 to August 2017, conventional suture was done in 119 patients, and during September 2017 to January 2019 quilting suture was done in 116 patients. Patients data such as age, BMI, medical history, familial history of cancer, TNM stage, and surgical method et al. were collected from electronic medical record. Institutional Ethical Review Board of Xiangya Hospital which abides to the Declaration of Helsinki.

### Surgical technique

**Fig. 1:**
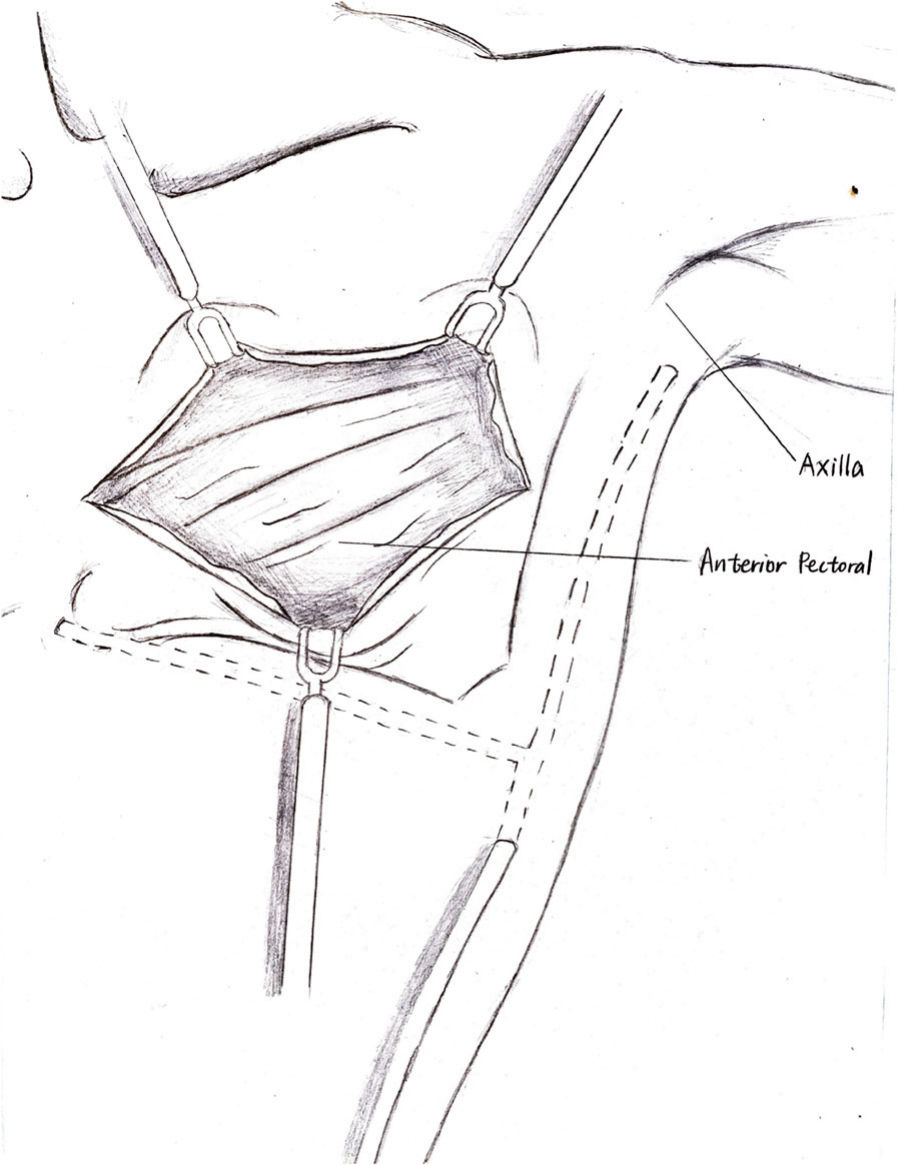
Illustration of conventional suture

**Fig. 2:**
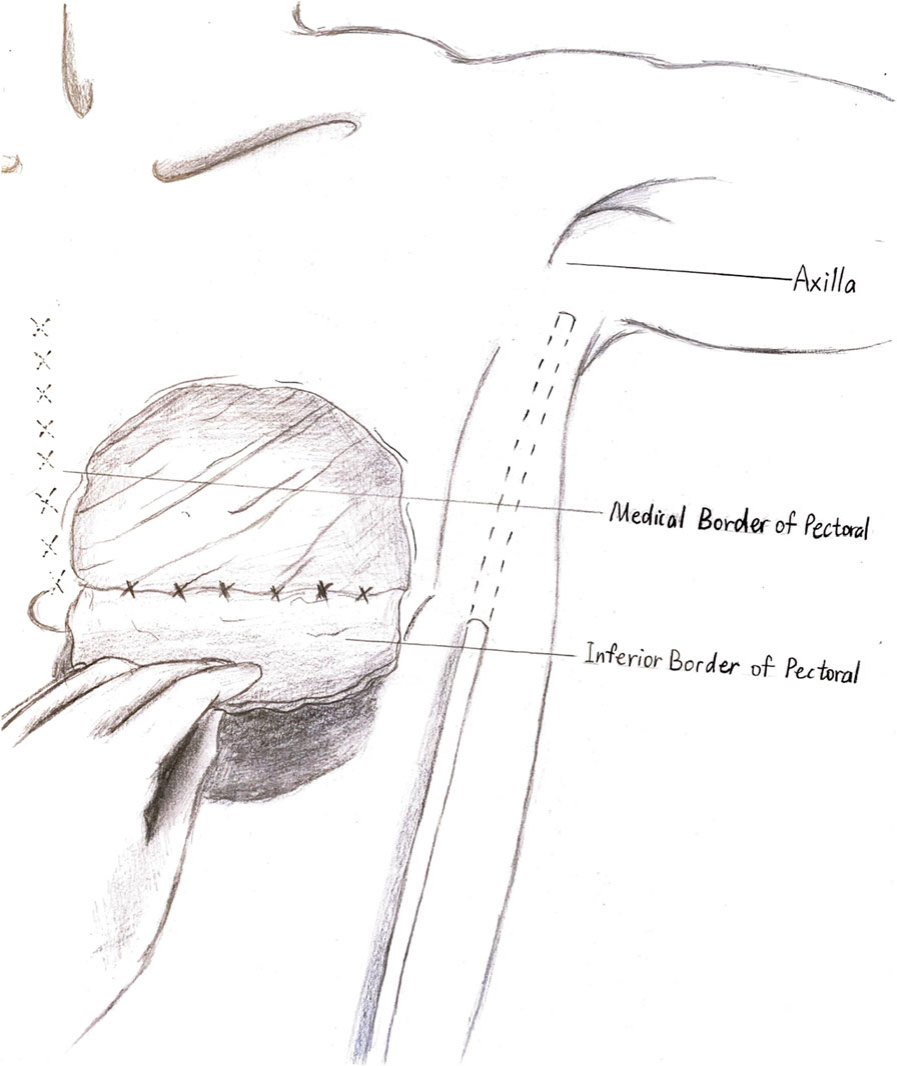
Illustration of quilting suture

The number of SLN was 2 to 5, and the ALND encompassed level I and II. All the patients had a closed suction drain in axillary area. For conventional wound suture, the skin flaps were not fixed subcutaneously but sutured at the edges; the second drain was inserted beneath the flaps near the medial and inferior border of the dead space at the pectoral area; and this drain was connected to the drain of axillary area subcutaneously as shown in Fig. 1. This pectoro-axillary drain converged and went through the lower lateral edge of skin graft and then connected to a sealed suction bottle. For quilting suture, the skin flaps were sutured to the underlying pectoralis major with 10–12 stitches in one row 1–2 cm away from the medial and inferior border of the dead space where were the most common places of seroma formation (Fig. 2).

For both suture techniques, the skin was closed at the edges as usual. The axillary area was not separated from the pectoral area and this would allow seroma to be drained out from the dead space of pectoral area. The daily drain output was measured and recorded. The drainage system was removed when the output was less than 20 ml over 24 h or on the day of discharge. Patients were discharged 5–9 days postoperatively after they had received the first cycle of chemotherapy if needed. The follow-up visit was recommended twice within 1 month after surgery, and physical and/or ultrasound examination were recommended during the visit.

### Outcomes

The primary outcome in this study was the incidence of Grade 2 or Grade 3 seroma in the pectoral area within 1 month after surgery. We applied the criteria for adverse events classification (CTCAE) 4.0 to categorize the seroma into Grade 1, Grade 2 and Grade 3. In short, Grade 1 seroma is asymptomatic and intervention is not indicated; Grade 2 and 3 are symptomatic and interventions are indicated. The secondary outcomes were the overall (Grade 1, 2 or 3) seroma rate in the pectoral area, rate of wound hematoma indicated with intervention of puncture or surgery, surgical site infection, inadequate wound healing, hospital stay after surgery (days), time for procedure of suturing flaps to pectoralis major plus inserting one drain in axilla in quilting group (seconds), time for inserting two drains in conventional group (seconds), and drainage volume (milliliters). We categorized seroma into early or late seroma according to the drainage removal time. Early seroma was defined as seroma formed before removal of drainage, and late seroma was defined as seroma formed after removal of drainage at any time but within 1 month postoperatively. Only the seroma at the pectoral area was recorded in this study.

### Statistical analysis

The characteristics of patients were compared between quilting and conventional suture group. Categorical variables were analyzed by χ2 test or Fisher exact test, and continuous variables were analyzed by *t* test or Wilcoxon rank-sum test. Statistical significance was defined at *p* value < 0.05. SPSS version 19.0 was used for statistical analysis [20].

## Results

### Patient characteristics

The study enrolled 116 patients in the quilting suture group without drainage of pectoral area and 119 patients in the conventional suture group with drainage of pectoral area. The patient characteristics were similar between the two groups, except for the familial history of cancer (Table 1).

**Table 1:** The patient characteristics were similar between the two groups, except for the familial history of cancer

		Conventional suture *n* = 119	Quilting suture *n* = 116	*P value*
Age (years)	Mean (SD)	50.8 (11.7)	52.7 (9.9)	0.205
BMI (kg/m^2^)	Mean (SD)	22.9 (2.8)	22.7 (2.9)	0.481
Hypertension	n(%)	11 (9.2)	20 (17.2)	0.070
Diabetes	n(%)	3 (2.5)	4 (3.4)	0.676
Smoking	n(%)	1 (0.8)	3 (2.6)	0.301
Menopause	n(%)	60 (50.4)	69 (59.5)	0.163
Familial history of cancer	n(%)	10 (8.4)	24 (20.7)	*0.007*
Neoadjuvant chemotherapy	n(%)	24 (20.2)	23 (19.8)	0.948
Surgery				
ME+SLN	n(%)	19 (16.0)	18 (15.5)	0.925
ME+ALND	n(%)	100 (84.0)	98 (84.5)	
TNM stage				
I	n(%)	27 (22.7)	37 (31.9)	0.235
II	n(%)	81 (68.1)	67 (57.8)	
III	n(%)	11 (9.2)	12 (10.3)	

*BMI* Body mass index (calculated as weight in kilograms divided by height in meters squared), *ME* Mastectomy, *SLN* Sentinel lymph node biopsy, *ALND* Axillary lymph node dissection, *SD* Standard deviation; Discrete variables used χ^2^ test or Fisher exact test; continuous variables *t* test or Wilcoxon rank-sum test

### Outcome comparisons

The incidence of Grade 2 and 3 seroma within 1 month after surgery was significantly higher in the conventional suture group compared with that in the quilting suture group (19.3% vs. 9.5%, *p* = 0.032). The rate of early seroma in Grade 2 and 3 was similar between the two groups (4.2% in conventional group vs. 5.2% in quilting group, *p* = 0.725); and the rate of late seroma in Grade 2 and 3 was significant higher in conventional group than that in quilting group (15.1% vs. 4.3%, *p* = 0.005). The incidence of Grade 1 seroma was significantly higher in quilting group compared with that in the conventional group (18.1% vs. 6.7%, *p* = 0.008). Overall, all grades of seroma in the pectoral area was comparable between the two groups (26.1% vs. 27.6%, *p* = 0.790) (Table 2).

**Table 2:** Outcome comparisons between quilting and conventional suture groups

		Conventional suture n = 119	Quilting suture n = 116	*P value*
Seroma (Grade 1, early)	n(%)	6 (5.0)	14 (12.1)	0.054
Seroma (Grade 2–3, early)	n(%)	5 (4.2)	6 (5.2)	0.725
Seroma (Grade 1, late)	n(%)	2 (1.7)	7 (6.0)	0.082
Seroma (Grade 2–3, late)	n(%)	18 (15.1)	5 (4.3)	*0.005*
Seroma (Grade 1)	n(%)	8 (6.7)	21 (18.1)	*0.008*
Seroma (Grade 2–3)	n(%)	23 (19.3)	11 (9.5)	*0.032*
Seroma	n(%)	31 (26.1)	32 (27.6)	0.790
Hematoma	n(%)	5 (4.2)	5 (4.3)	0.967
Surgical site infection	n(%)	5 (4.3)	4 (3.4)	0.764
Inadequate wound healing	n(%)	2 (1.7)	2 (1.7)	0.979

Discrete variables used χ^2^ test or Fisher exact test

The incidence of Grade 2 and 3 seroma or late seroma in Grade 2 and 3 was significant higher in the conventional suture group compared with that in the quilting group (*p* = 0.032 and *p* = 0.005 respectively). The incidence of Grade 1 seroma was significantly higher in quilting group compared with that in the conventional group (*p* = 0.008).

There were no significant differences between the two groups regarding hematoma, surgical site infection, inadequate wound healing and length of hospital stay after surgery (Tables 2 and 3).

**Table 3:** Outcome comparisons between quilting and conventional suture groups

		Conventional suture n = 119	Quilting suture n = 116	*P value*
Hospital stay after surgery (days)	Mean (SD)	7.8 (1.0)	7.8 (1.0)	0.930
Time for suture (seconds)	Mean (SD)	109.1 (9.1)	504.7 (102.6)	*< 0.001*
Drain volume (ml)	Mean (SD)	520.5 (84.3)	374.9 (57.1)	*< 0.001*

*SD* Standard deviation; Continuous variables used *t* test or Wilcoxon rank-sum test

Quilting suture was significantly associated with longer time of surgery procedure and less volume of drainage compared with that of conventional suture (*p* < 0.001 both).

Quilting suture was significantly associated with longer operation time [mean (SD), 504.7 s (102.6 s)] for suturing flaps to pectoralis major compared with conventional suture [mean (SD), 109.1 s (9.1 s)] (*p* < 0.001). Patients treated with quilting suture were more likely to experience less volume of drainage than those with conventional suture (374.9 vs. 520.5 ml, *p* < 0.001) as shown in the Table 3.

### Risk factors

A multivariate logistic regression was used to evaluate the risk factors of seroma formation as shown in Table 4. Old age and high BMI were found to be risk factors for Grade 2 and 3 seroma, in both early and late stage. Patients younger than 60-year-old or with BMI less than 25 experienced much less Grade 2 and 3 seroma within 1 month postoperatively when compared to patients older than 60 or with BMI higher than 25 (*p* < 0.05). Quilting suture had the potential to reduce the incidence of Grade 2 and 3 seroma in the late stage, and a multivariate analysis showed quilting suture was significantly associated with less seroma (95% CI = 0.068–0.719, *P* = 0.012). Hypertension, diabetes, neoadjuvant chemotherapy and type of surgery had no influence on developing a Grade 2 and 3 seroma in the pectoral area.

**Table 4:** A multivariate logistic regression of the risk factors of seroma (Grade 2–3)

Parameter	Seroma (Grade 2–3, early)		Seroma (Grade 2–3, late)	
	OR (95% CI)	*p* value	OR (95% CI)	*p* value
Age (year) (≥60 vs < 60)	1.015–1.186	*0.019*	1.006–1.114	*0.029*
BMI (kg/m^2^) (≥25 vs < 25)	1.176–2.097	*0.002*	1.217–1.795	*< 0.001*
Hypertension	0.084–2.116	0.423	0.158–2.449	0.497
Diabetes	0.078–128.994	0.541	0.022–2.015	0.176
Neoadjuvant chemotherapy	0.072–24.505	0.847	0.170–3.468	0.732
ME+ALND (Yes vs No)	0.129–11.509	0.864	0.056–2.177	0.259
Quilting (Yes vs No)	0.342–6.649	0.588	0.068–0.719	*0.012*

*ME* Mastectomy, *SLN* Sentinel lymph node biopsy, *BMI* Body mass index

Old age and high BMI were independent risk factors for early and late seroma in Grade 2–3 (*p* < 0.05 both). Quilting suture was protective factor for late seroma formation in Grade 2–3 (*p* < 0.05).

## Discussion

This study showed that quilting suture was associated with lower incidence of Grade 2 and 3 seroma compared with that of conventional suture within 1 month after mastectomy with SLN or ALND, especially for the late seroma in Grade 2 and 3. This study also showed that old age and high BMI were risk factors on postoperative seroma formation, consistent with previous results from similar studies [9, 11, 21–23].

In this study, the incidence of seroma of all grades in the pectoral area was similar between quilting suture and conventional suture groups, while the incidence of Grade 2 and 3 seroma was significantly higher in conventional suture group. This was in line with some other studies which showed that quilting suture procedure could indeed reduce the incidence of seroma formation, especially the clinically significant seroma [11, 19, 24, 25]. To our knowledge, there was no need of any interventions for Grade 1 seroma, so even though there were more events of Grade 1 seroma happening in quilting suture group, patients only needed observation and followed the instruction to avoid excessive exercise. But for Grade 2 and 3 seroma, repeated aspiration and irregular clinic visits were suggested for these patients, which increased the medical cost and delayed adjuvant therapies. In rare cases, a persistent seroma was resistant to conservative treatment and required drain reinsertion and even surgical resection of fibrous capsule wall [26–28]. In our cohort, we categorized seroma into early or late stage seroma according to removal time of drainage, and we found that the incidence of early seroma in Grade 2 and 3 was comparable between the two groups (4.2% in conventional group vs. 5.2% in quilting group). After the removal of drainage, the incidence of new cases of Grade 2 and 3 late stage seroma was low in the quilting suture group, which was 4.3% compared with 15.1% in the conventional suture group. The high incidence of late seroma in Grade 2 and 3 contributed to the significant difference between the two groups when we analyzed Grade 2 and 3 seroma within 1 month postoperatively (19.3% in the conventional suture vs. 9.5% in the quilting suture, *p* = 0.032).

The quilting suture which fixes the skin flaps to the subcutaneously tissue can remarkably reduce the formation of late seroma in Grade 2 and 3, while has no influence on the early seroma in Grade 2 and 3. This implied that prolonged drainage might be a solution for the Grade 2 and 3 seroma at late stage in conventional suture group, albeit this would be associated with patients discomfort, increased infection, shoulder immobilization and longer hospital stay [29–31]. In our institution patients would receive the adjuvant chemotherapy, if needed, 7 days after surgery and then get discharged with or without drain, so the prolonged drainage will not influence the hospital stay. Table 3 showed that hospital stay after surgery was similar between the two groups (*p* = 0.930).

In our study, we recorded the time for fixing skin flaps to subcutaneous tissue plus inserting one drain in axilla in quilting suture group, and the time for inserting two drains in the conventional suture group. We found quilting suture was significantly associated with longer operation time compared with that in conventional suture (504.7 s vs. 109.1 s, *p* < 0.001), with an increase of around 7 min. This findings are supported by that of Ten Wolde et al., that quilting suture was applied after axillary lymph node dissection and mastectomy, and it required approximately 10 to 20 more minutes to operate than the conventional way [11]. Another study conducted by Ouldamer et al. also using quilting suture to reduce seroma at pectoral area showed no significant difference concerning the operation time between the two suture methods [12]. However, they recorded the operation time from skin incision to the end of wound closure, instead of only for fixing skin flaps to pectoralis major and insertion of drains as what we did. Nevertheless, we noticed that the average operative time was 8 min longer in quilting suture group compared with that in conventional closure group in Ouldamer’s study [12]. In our opinion, the time for quilting suture could be shortened if we applied running suture to fix skin flaps to subcutaneous tissue in the future, other than interrupted suture in this study. It is recommended to apply quilting suture technique to reduce the incidence of seroma, repeated aspiration and irregular clinic visit, with only 5 to 7 min longer operation time.

Our aim in this study was similar to the objective of Ouldamer et al. who compared quilting suture of the dead space of the pectoral area with conventional closure, on seroma formation in women treated with mastectomy, but there were some differences in details [12]. First, we did the quilting suture only in one row of interrupted suture, 10–12 stitches, 1–2 cm away from the medial and inferior border of the dead space. In the Ouldamer’s study, several parallel rows of evenly spaced running sutures were applied to fix the skin flaps to the underlying muscle. Second, we did not close the axillary area to separate it from pectoral area. This allowed to drain out liquid at the lateral border of pectoral area, but not liquid at the medial and inferior border of pectoral area. Third, we converged the two drains into one tube to go through skin flap with only one suction bottle in the conventional suture, which reduced patients’ discomfort and lowered the incidence of infection. Furthermore, we noticed that quilting suture could significantly reduce the incidence of late seroma in Grade 2 and 3, but not early seroma in Grade 2 and 3.

There are some limitations in our study. This was a retrospective study without randomization. The observers who performed the follow-up examination were not blind to the suture procedures. A prospective multicenter study with large number of patients is needed to prove the efficacy of quilting suture.

## Conclusion

In conclusion, old age, high BMI and conventional suture are important prognostic factors influencing pectoral seroma formation in breast cancer patients undergoing mastectomy. Quilting suture could remarkably decrease the rate of Grade 2 and 3 seroma at pectoral area within 1 month after mastectomy, especially the late seroma in Grade 2 and 3.

## Data Availability

The datasets generated and/or analysed during the current study are not publicly available due to its usage for another article, but are available from the corresponding author on reasonable request.

## Abbreviations

ME: Mastectomy
SLN: Sentinel lymph nodes biopsy
ALND: Axillary lymph nodes dissection
BMI: Body mass index
